# Expression of Dual-Specificity Phosphatase 5 Pseudogene 1 (DUSP5P1) in Tumor Cells

**DOI:** 10.1371/journal.pone.0089577

**Published:** 2014-02-24

**Authors:** Martin S. Staege, Katja Müller, Stefanie Kewitz, Ines Volkmer, Christine Mauz-Körholz, Toralf Bernig, Dieter Körholz

**Affiliations:** Department of Pediatrics, Martin Luther University Halle-Wittenberg, Halle, Germany; University of Birmingham, United Kingdom

## Abstract

Sequencing of individual clones from a newly established cDNA library from the chemoresistant Hodgkin's lymphoma cell line L-1236 led to the isolation of a cDNA clone corresponding to a short sequence from chromosome 1. Reverse transcriptase-polymerase chain reaction indicated high expression of this sequence in Hodgkin's lymphoma derived cell lines but not in normal blood cells. Further characterization of this sequence and the surrounding genomic DNA revealed that this sequence is part of a human endogenous retrovirus locus. The sequence of this endogenous retrovirus is interrupted by a pseudogene of the dual specificity phosphatase 5 (DUSP5). Reverse transcriptase-polymerase chain reaction revealed high expression of this pseudogene (DUSP5P1) in HL cell lines but not in normal blood cells or Epstein-Barr virus-immortalized B cells. Cells from other tumor types (Burkitt's lymphoma, leukemia, neuroblastoma, Ewing sarcoma) also showed a higher DUSP5P1/DUSP5 ratio than normal cells. Furthermore, we observed that higher expression of DUSP5 in relation to DUSP5P1 correlated with the expression of the pro-apoptotic factor B cell leukemia/lymphoma 2-like 11 (BCL2L11) in peripheral blood cells and HL cells. Knock-down of DUSP5 in HL cells resulted in down-regulation of BCL2L11. Thus, the DUSP5/DUSP5P1 system could be responsible for regulation of BCL2L11 leading to inhibition of apoptosis in these tumor cells.

## Introduction

The prognosis for patients with Hodgkin's lymphoma (HL) has been improved constantly during the last decades [1]. Nevertheless, some patients cannot be cured with currently established therapy protocols. On the other hand, toxicity of applied chemo- and radiotherapy is high and this treatment is associated with a risk of late complications including secondary malignancies, infertility, breastfeeding problems or cardiovascular diseases [2]–[5]. Therefore, the recognition of new prognostic factors for patients with HL is desirable [6]. The characterization of factors associated with resistant disease may lead to the identification of alternative therapeutic strategies. *Vice versa*, the identification of such factors might also allow the reduction of toxic treatment elements for patients with favorable disease characteristics.

Using DNA microarray analysis, we characterized the gene expression profile of HL cell lines with different sensitivity for cytotoxic drugs and identified transcripts which are present only in resistant cells [7]. DNA microarrays allow the simultaneous detection of the complete transcriptome of tumor cells and control samples in a single experiment [8]. However, this method did not allow the screening for unknown transcripts and did not allow the direct functional analysis of tumor relevant transcripts. One elegant method for the identification of cDNAs corresponding to transcripts of interest is based on the transfection of cDNA libraries into eukaryotic cells and functional selection of transfected cell [9]. For the functional screening of cDNAs encoding HL derived transcripts with involvement in disease pathology we established cDNA libraries from different established HL cell lines. During initial characterization of such a cDNA library from HL-cell line L-1236, we detected unusual short transcripts with homology to endogenous retroviruses. A large number of human endogenous retroviruses (ERV) have been identified [10] and activation of these ERV has been implicated in autoimmunity, neurodegenerative diseases and cancer [11]–[13]. ERV-related sequences form approximately 10% of the human genome. ERV loci are derived from exogenous retroviruses that were stably integrated during evolution in the host genome and are transmitted via the germ line. The majority of ERV have been inactivated by deletions, insertions, or other mutations. Only few ERV contain intact open reading frames for one or more viral genes. Recently, activation of ERV sequences in HL has been observed as a mechanism for aberrant gene expression [14]. Here, we describe the expression of another ERV-associated locus in HL cells. Our observation adds further evidence for the activation of endogenous retroviruses in these tumor cells. Expression of the newly identified ERV in HL cells is closely linked to expression of a pseudogene of the dual specificity phosphatase 5 (DUSP5). DUSP5 is involved in negative regulation of the extracellular signal-regulated kinase (ERK) pathway [15]. Interference of transcripts from the DUSP5 pseudogene locus with DUSP5 activity might influence this important signaling pathway.

## Materials and Methods

### Ethics Statement

Peripheral blood mononuclear cells (PBMC) from anonymous healthy donors and patients with HL were isolated with written informed consent and approval by the ethics committee of the Medical Faculty of the Martin Luther University Halle-Wittenberg.

### Cells and cell culture

HL cell lines L-1236 [16], L-540 [17], L-428 [18], HDLM-2 [18], and KM-H2 [19] were obtained from the Deutsche Sammlung für Mikroorganismen und Zellkulturen (Braunschweig, Germany). Lymphoblastoid cell lines were established as described [20]. All cells were cultured in RPMI1640 medium supplemented with 10% fetal calf serum and penicillin/streptomycin. Peripheral blood mononuclear cells (PBMC) from healthy donors and patients with HL were isolated as described [21].

### Generation of a cDNA library from L-1236 cells

Total RNA was isolated from cell line L-1236 by using Trizol reagent (Invitrogen, Karlsruhe, Germany) following the manufacturer's protocol. Thereafter, mRNA was enriched starting from 650 µg of total RNA by using the µMACS mRNA isolation kit (Miltenyi, Bergisch-Gladbach, Germany). The cDNA library was generated by using the pCMV-Script XR cDNA library construction kit (Agilent Technologies, La Jolla, CA, USA) with slight modifications. In short, 4.5 µg mRNA was subjected to first strand cDNA synthesis in a total volume of 45 µl for 1 hour at 42°C. After addition of 20 µl 10× second strand buffer, 6 µl second strand dNTP mixture, 116 µl sterile water, 2 µl RNase H (1.5 U/µl), and 11 µl DNA polymerase I (9 U/µl), second strand synthesis was performed at 16°C for 2.5 hours. Blunt ending and adapter ligation was performed according to manufacturer's instructions. After digestion with *EcoR*1, phosphorylation of *EcoR*1 ends and digestion with *Xho*I, cDNA was fractionated by sepharose CL-2B gel filtration. Fractions with high cDNA concentration and the expected cDNA size were ligated into the vector pCMV-Script. After ligation, vectors were transformed into XL10-Gold ultracompetent cells and the primary library was amplified at 30°C. After plating on agarose plates, random clones were analyzed by restriction digest with *Not*I.

### RT-PCR

RNA from HL cell lines, LCL, PBMC, and additional tumor cell lines was isolated by using Trizol reagent (Invitrogen, Karlsruhe, Germany) following the manufacturer's protocol. For reverse transcription the following enzymes have been used: (i) SuperScript II RT (Invitrogen), (ii) M-MLV RT, RNase H(-) point mutant (Promega, Mannheim, Germany), and (iii) RevertAid H Minus RT (Fermentas, St. Leon-Rot, Germany). After reverse transcription of 2 µg of RNA, PCR was performed as described elsewhere [7]. Quantitative RT-PCR was performed as described [22]. The following primer combinations have been used: actin beta (ACTB): 5′-ggc atc gtg atg gac tcc g-3′ and 5′-gct gga agg tgg aca gcg a-3′; B cell lymphoma/leukemia 2-like 11 (BCL2L11): 5′-cat cgc ggt att cgg ttc-3′ and 5′-cct tct cgg tca cac tca ga-3′; dual specificity phosphatase 5 (DUSP5): 5′-acc tac cct gag gtc cgt ct-3′ and 5′-gga ggc ctt cga tta cat ca-3′; DUSP5 pseudogene 1 (DUSP5P1): 5′-gtg ctg aac tag ggg agc tg-3′ and 5′-aga tgg tgg gtg aac agg ag-3′; glyceraldehyde-3-phosphate dehydrogenase (GAPDH): 5′-cca tgg aga agg ctg ggg-3′ and 5′-caa agt tgt cat gga tga cc-3′; ERVK_1q42.13_library: 5′-tca act acc aag tgc ttg g-3′ and 5′-ttt agt tat tca atc tca tgt aat ta-3′; ERVK_1q42.13: 5′-tga att cat gaa att gct aat aag a-3′ and 5′-gga ttg ggg gaa cct aga aa-3′. For determination of PCR efficiency, target sequences were amplified by conventional PCR using primer combinations 5′-gtg ctg aac tag ggg agc tg-3′ and 5′-aga tgg tgg gtg aac agg ag-3′ (DUSP5), 5′-gca atc aga ttc ata gaa aga act c-3′ and 5′-ctt caa cca act gcc tcc at-3′ (DUSP5P1), 5′-cca tgg aga agg ctg ggg-3′ and 5′-caa agt tgt cat gga tga cc-3′ (GAPDH), or 5′-cat cgc ggt att cgg ttc-3′ and 5′-cct tct cgg tca cac tca ga-3′ (BCL2L11), respectively. PCR products were cloned into vector pGEM-T Easy (Promega) and sequenced as described [22]. Equal amounts of vectors were digested with *Sal*I, serially diluted 1∶10 and used as templates for quantitative PCR. A typical result is shown in Figure S1. Relative expression values were calculated using standard 2^−ΔΔct^ method [23]. In addition, molar concentrations of transcripts in relation to the concentration of the house keeping control GAPDH were calculated on the basis of the standard curves (Figure S1).

### Sequence analysis

Sequencing of pCMV-Script vector inserts was performed as described [22] by using the following pCMV-Script specific primers: 5′-aat taa ccc tca cta aag gg-3′ and 5′-taa tac gac tca cta tag gg-3′. The sequence of the ERVK_1q42.13 insert was submitted to GenBank (accession number JZ534323). Sequence analysis was performed with BLAST [24] and RetroSearch [25]. For multiple sequence alignments, CLUSTALW [26] was used. *In silico* promoter analysis and protein structure prediction was performed with NNPP2.2 [27] and SWISS-MODEL [28], respectively. For secondary structure prediction the structure of dual-specificity phosphatese 6 (DUSP6) [29] was used as template.

### Knock-down of DUSP5 and microarray analysis

Vector-based knock-down of DUSP5 in L428-cells was performed by using the BLOCK-iT POL II miR RNAi expression vector kit with EmGFP (Invitrogen, Karlsruhe, Germany) according to manufacturer's instructions. For this end, the two oligonucleotides 5′-TGC TGA TGG TAG GCA CTT CCA AGG GTA GTT TTG GCC ACT GAC TGA CTA CCT TGG GTG CCTA CCA T-3′ (top strand) and 5′-CCT GAT GGT AGG CAC CCA AGG TAG TCA GTC AGT GGC CAA AAC TAC CTT GGA AGT GCC TAC CAT C-3′ (bottom strand) were annealed and cloned into the vector pcDNA6.2-GW/EmGFP-miR. After transfection of L-428 cells with this vector or the empty control vector, global gene expression was analyzed by using Affymetrix HG_133A microarrays essentially as described [7]. Cel files were processed with Affymetrix Expression Console using the MicroarraySuite 5.0 algorithm and scaled to the same target intensity of 500. DNA microarray data have been submitted to the Gene Expression Omnibus (GEO) data base (accession number GSE52831).

## Results

### Identification of endogenous retrovirus transcripts in Hodgkin's lymphoma cell lines

**Figure 1:**
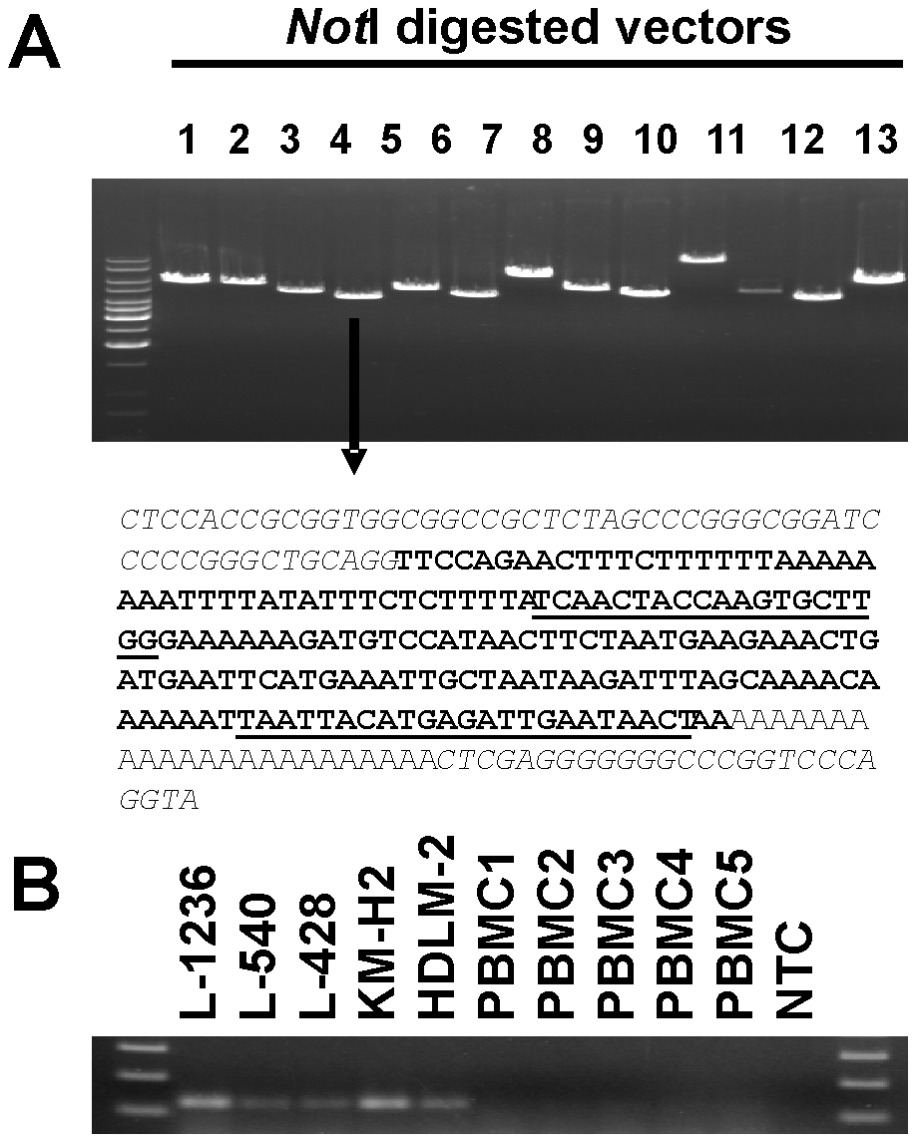
Identification of ERV expression in HL cell lines. (A) 13 individual vectors from a cDNA library from HL cell line L-1236 were digested with *Not*I, analyzed by gel electrophoresis, and sequenced as described in Material and Methods. The sequence of the insert from the shortest vector (arrow) is presented. The vector sequence is printed in italics; the insert sequence that is also present in the human genome is presented in bold type. Binding sites for the primers used in panel B are underlined. (B) Presented are results from a RT-PCR with primers (ERVK_1q42.13_library) with specificity for the sequence from panel A. cDNA from HL cell lines and normal PBMC was used as template for PCR. NTC: no template control.

**Figure 2:**
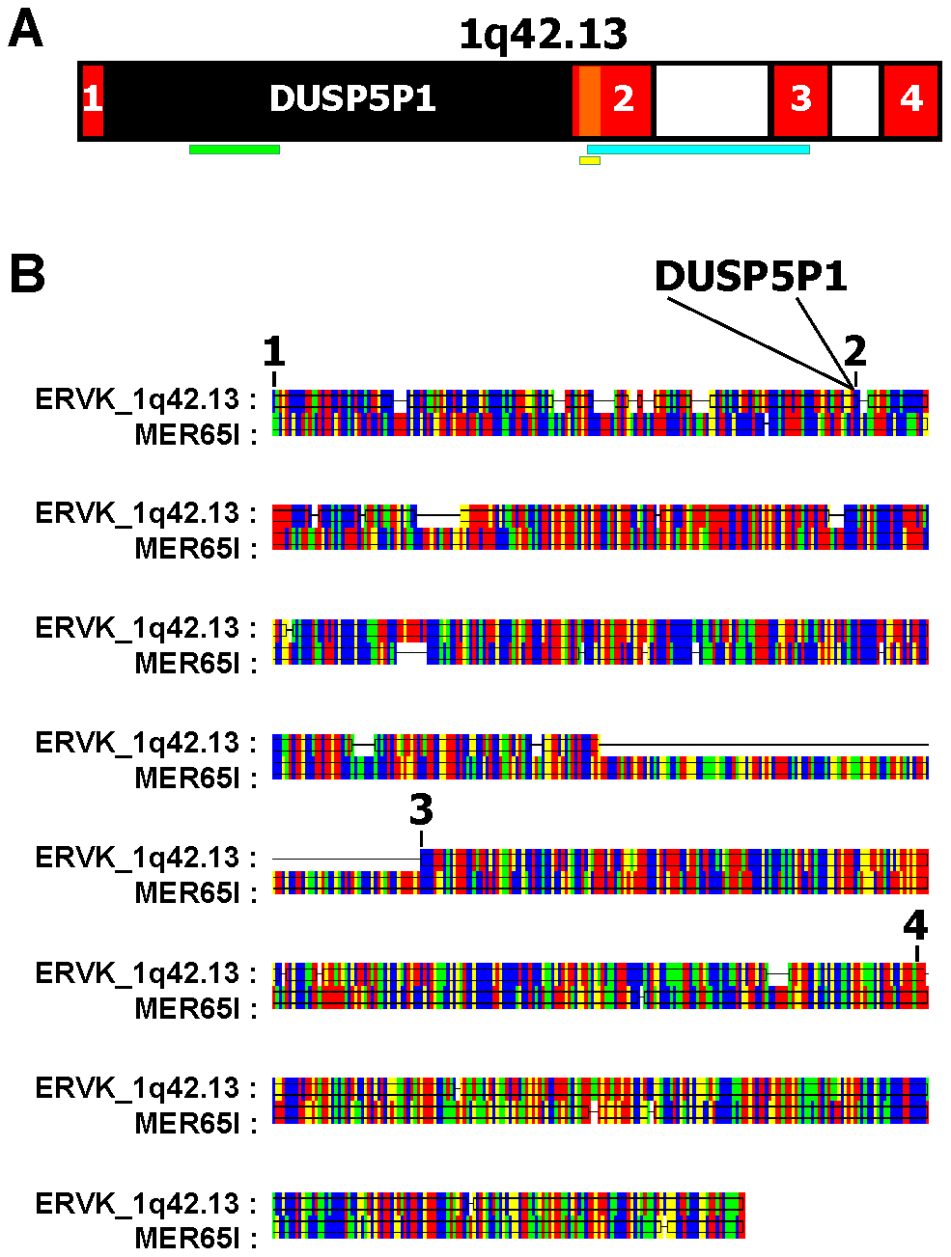
Schematic presentation of human chromosome 1 band 1q42.13 and the DUSP5P1 locus. (A) The sequence of the vector shown in Figure 1 is indicated by an orange rectangle. This sequence is part of a larger ERV sequence (colored red) on chromosome 1 at band 1q42.13 which was identified as described in Material and Methods. This larger sequence is split into several parts. Only the 4 parts with highest homology to an ERV in chromosome 4 are shown. Part one and two of this ERV are separated by a pseudogene of DUSP5 (DUSP5P1). Locations of PCR Products with primers with specificity for DUSP5P1 (green), the cloned cDNA (ERVK_1q42.13_library; yellow) and the ERV segments 2 and 3 (ERVK_1q42.13; blue) are indicated by horizontal bars. (B) Sequence alignment of the newly identified ERV and an ERV from chromosome 4 (MER65I, RetroSearch ERV-ID: 16168). The position of the DUSP5P1 insertion in ERVK_1q42.13 is indicated. Color code: red: A; green: C; yellow: G; blue: T. Data visualization was performed with GeneDoc (http://www.psc.edu/biomed/genedoc).

We established a cDNA library from HL cell line L-1236. After ligation of cDNA fractions with the cloning vector, individual ligation reactions were transformed in *E. coli* and plated on agar plates for determination of ligation efficiency. Individual *E. coli* colonies were arbitrarily chosen for further characterization of the transformed vectors. Vectors were isolated, digested (linearized) with *Not*I, and analyzed on agarose gels. As shown in Figure 1A, individual vectors carried different cDNA inserts as revealed by different sizes of the linearized vectors. Individual vectors were sequenced by using primers flanking the cloning site. We found cDNA inserts of varying length in all vectors. A very short insert of 192 base pairs (bp) with no long open reading frame was found in one of the smallest vectors (Figure 1A). We designed primers with specificity for this insert and tested the presence of the corresponding transcripts in HL cell lines. We detected expression of this sequence in all HL cell lines tested but not in normal PBMCs (Figure 1B). A BLAST analysis indicated that this sequence was derived from chromosome 1 in close proximity to the 5′ end of a locus encoding a dual specificity phosphatase 5 pseudogene (DUSP5P1). A RetroSearch analysis indicated that the cloned sequence had high similarity to endogenous retroviruses (ERV). The highest similarity was found to an ERV from chromosome 4 (RetroSearch ID: 16168). We used the complete sequence of this ERV (MER65I) as query in a BLAST search. The result revealed that the ERV from the DUSP5P1 locus on chromosome 1 is split into multiple segments from which two segments surround the DUSP5P1 locus (Figure 2A and B). Based on the chromosomal location and similarity with other ERV sequences from ERV family K, in the following sections we use the name ERVK_1q42.13 for this ERV.

### Expression of DUSP5P1 and ERVK_1q42.13 in HL cells

**Figure 3:**
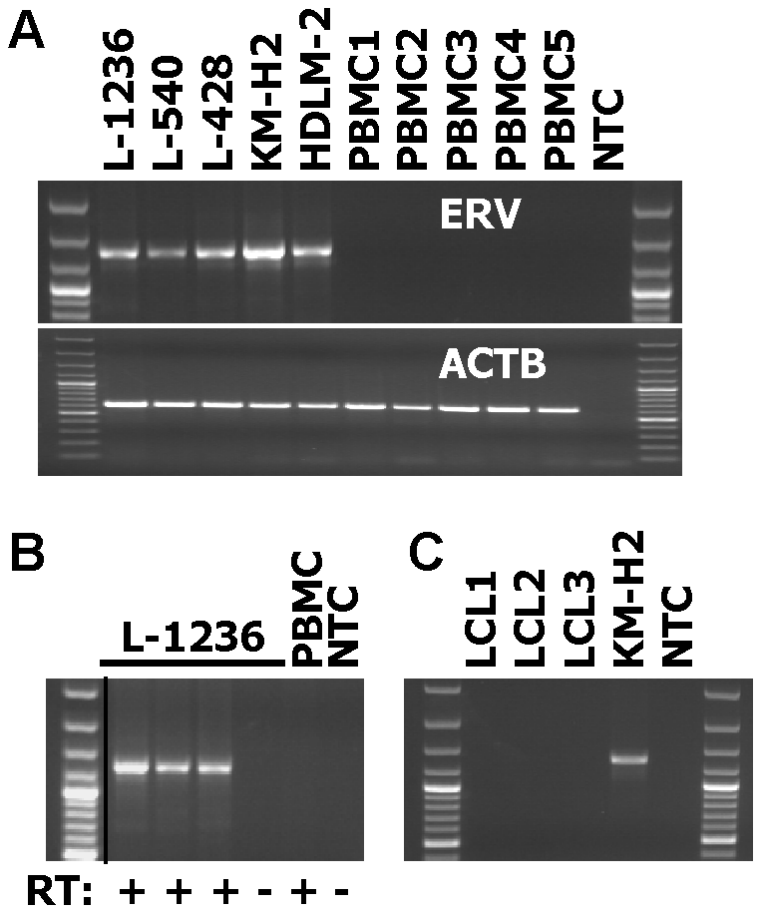
Expression of ERVK_1q42.13 in HL cell lines. (A) Presented are results from a RT-PCR with primers with specificity for actin beta (ACTB) and ERVK_1q42.13. cDNA from HL cell lines and normal PBMC was used as template for PCR. NTC: no template control. (B) Presented are results from a RT-PCR with primers with specificity for ERVK_1q42.13. cDNA from HL cell line L-1236 and normal PBMC was used as template for PCR. cDNA was synthesized by using reverse transcriptase (RT:+) from three different vendors (see Material and methods). In addition, RNA was used without reverse transcription (RT:-). NTC: no template control. The black dividing line indicates removal of five irrelevant lanes from the original image. (C) Presented are results from a RT-PCR with primers with specificity for ERVK_1q42.13. cDNA from HL cell line KM-H2 and three different lymphoblastoid cell lines (LCL) was used as template for PCR. NTC: no template control.

**Figure 4:**
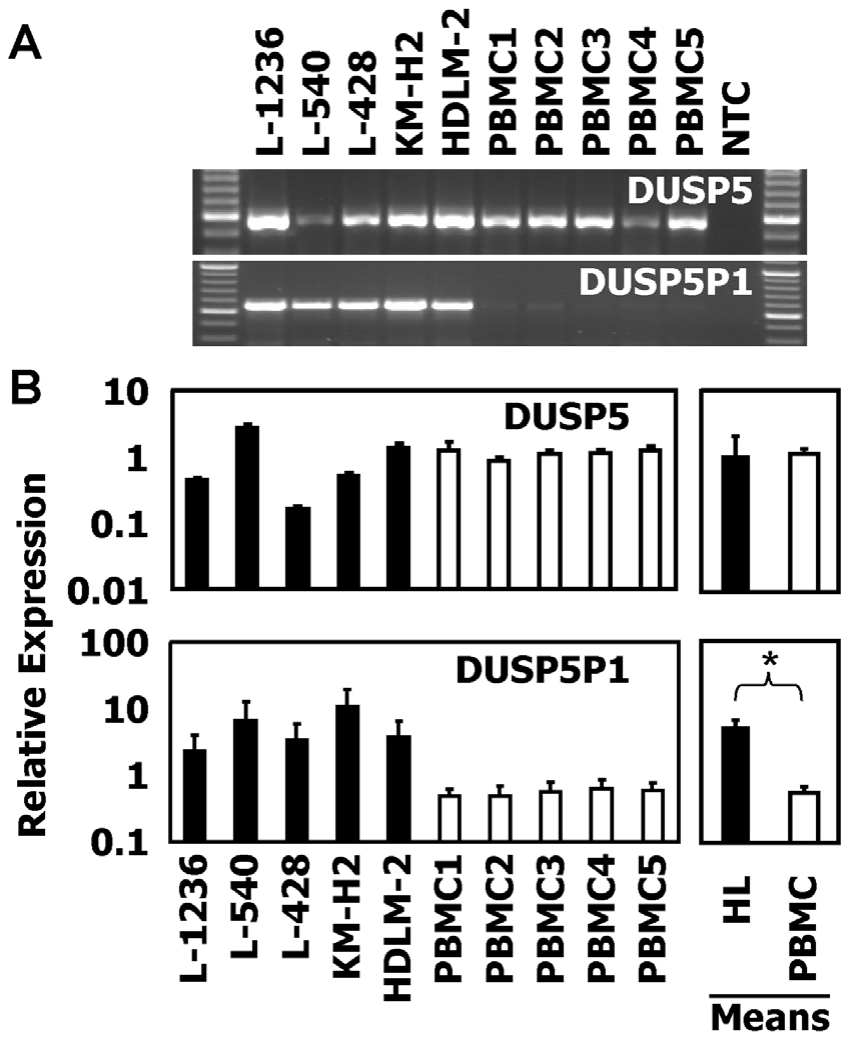
Expression of DUSP5P1 and DUSP5 in HL cell lines. (A) Presented are results from a RT-PCR with primers with specificity for DUSP5 and DUSP5P1. cDNA from HL cell lines and normal PBMC was used as template for PCR. NTC: no template control. (B) Presented are results from a quantitative RT-PCR (means and standard deviations from 3 experiments) with primers with specificity for DUSP5 and DUSP5P1. cDNA from HL cell lines and normal PBMC was used as template for PCR. For calculation of relative expression values, GAPDH was used as housekeeping control and the mean Δct value was set as 1. In addition to the results from individual cell lines, the mean values from HL cell lines and PBMC are presented. Asterisk indicate statistical significance (p<0.05; Student's t-test).

We asked whether the DUSP5P1 gene and additional segments of the ERV were also expressed in HL cells. For this end, we designed additional primers corresponding to segments 2 and 3 of ERVK_1q42.13 (see Figure 2). As shown in Figure 3A, in all HL cell lines investigated we found expression of ERVK_1q42.13. Normal PBMCs showed no signals. Reverse transcription with different reverse transcriptase enzymes led to identical results but we obtained no signals in HL cell lines without reverse transcription (Figure 3B), excluding contamination with genomic DNA. Expression of ERVK_1q42.13 was independent of the B cell phenotype of the HL cells as indicated by the expression of ERVK_1q42.13 in HL cells with a T cell phenotype (L-540, HDLM-2). In addition, expression of ERVK_1q42.13 was not detectable in Epstein-Barr virus-immortalized B cells (LCL, Figure 3C). In contrast to DUSP5P1, DUSP5 was detectable by conventional RT-PCR (Figure 4A) and quantitative RT-PCR (Figure 4B) both in HL cell lines and normal PBMCs. In all samples the molar concentrations of DUSP5 exceeded the concentrations of DUSP5P1 (Figure S2). High signals for DUSP5P1 were also detected in DNA microarray data from HL cell lines and microdissected HL cells (microarray data from the Gene Expression Omnibus (GEO) data base; Figure S3).

**Figure 5:**
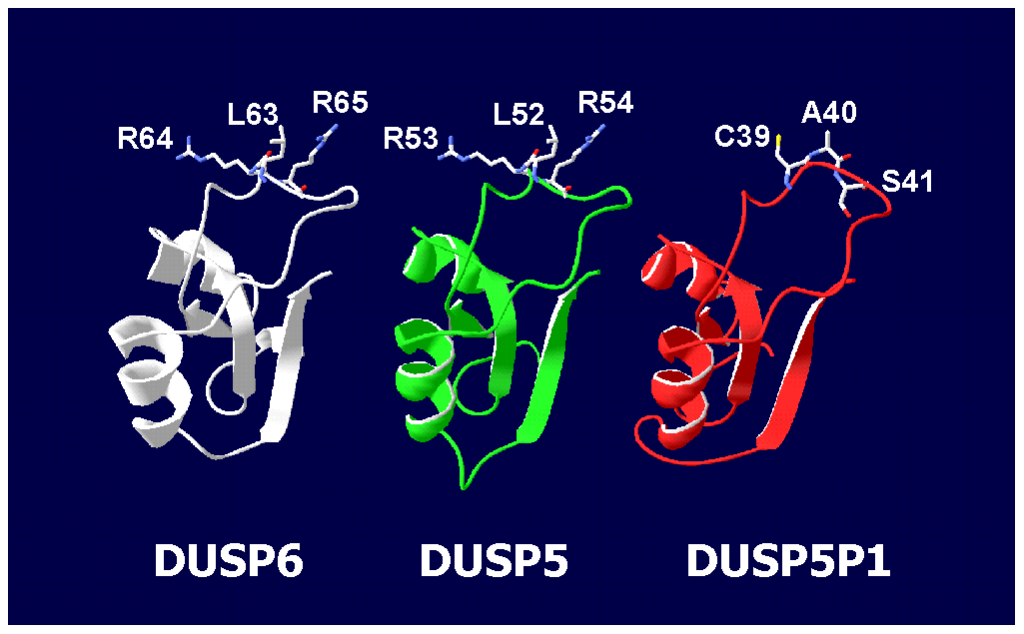
Homology modeling of a putative DUSP5P1 derived polypeptide. Presented is the result from an *in silico* homology modeling experiment using the substrate binding domain from DUSP6 [29] as template. The putative DUSP5P1 peptide (red) was predicted on the basis of a promoter scan followed by translation of all possible reading frames. This peptide (MLRKEAAAGW MVLGCRPYLA FTALSVPGSL NINLYSLVCA SPGRLWGQRA TCCQMPRSTL LLQEGSILAA VMVLN) is derived from the first open reading frame after the predicted transcription start site. In addition, the structure of the homologue region of DUSP5 (green) was predicted by using DUSP6 (white) as template. For better visibility, only the sequences from DUSP6 and DUSP5 corresponding to the predicted DUSP5P1 peptide are shown. Amino acids important for substrate binding by DUSP6 and the corresponding amino acids from DUSP5 and DUSP5P1 are highlighted.

Using the DUSP5 RNA sequence as query for a BLAST search, we identified 3 additional processed pseudogenes in the human genome (Figure S4), suggesting that DUSP5-like sequences were repeatedly involved in gene rearrangements during evolution. *In silico* promoter prediction indicated that transcription of DUSP5P1 most likely starts 44 base pairs up-stream of the sequence with high homology to DUSP5. Such transcripts allow the translation of a polypeptide corresponding in part to the substrate binding domain of DUSP5 surrounding the putative substrate binding site (Figure S5). In DUSP5 this binding site is characterized by two arginine residues and is highly conserved in vertebrates. Interestingly, these and the following amino acids are mutated in DUSP5P1 (Figure S5). Figure 5 shows results from homology modeling of this peptide using the structure of the mitogen-activated protein kinase 1-binding domain of DUSP6 [29] as template.

### Expression of DUSP5P1 and DUSP5 in tumor cells

**Figure 6:**
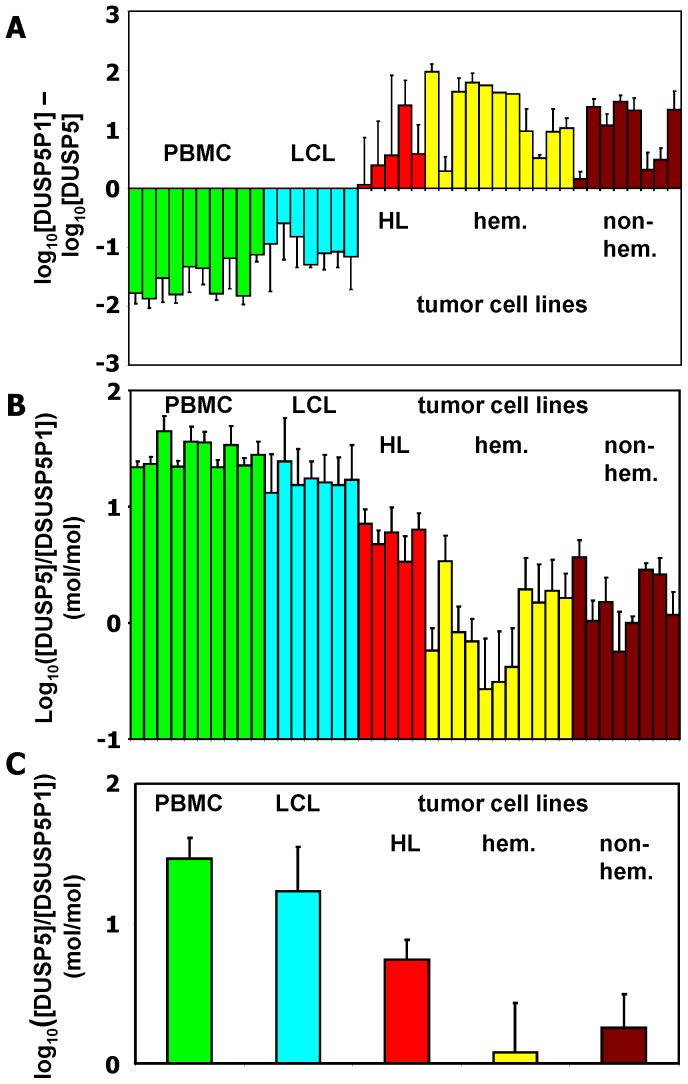
The ratio of DUSP5 and DUSP5P1 discriminates between malignant and non-malignant cells. Quantitative RT-PCR was used for determination of expression of DUSP5 and DUSP5P1 in cell lines and normal PBMC. (A) For calculation of relative expression values, GAPDH was used as housekeeping control and the mean Δct value was set as 1. Presented are the DUSP5P1/DUSP5 ratios in the indicated samples (from left to right: 10 PBMC, 7 EBV immortalized B cell lines (LCL), 5 HL cell lines (L-1236, L-428, L-540, KM-H2, HDLM-2), hematopoietic (hem.) tumor cell lines (Daudi, Raji, Jurkat, THP-1, 697, cALL2, NALM-6, Kasumi, K562, HL-60, U937), 4 neuroblastoma cell lines (SiMa, Kelly, SH-Sy5y, CHP-134), 4 Ewing sarcoma cell lines (SK-N-MC, A673, RD-ES, TC71). (B) Absolute copy numbers of DUSP5 and DUSP5P1 were calculated based on the vector titrations (see Figure S1). Presented are the DUSP5/DUSP5P1 copy numbers ratios (means and standard deviations) in the indicated samples (same samples as in panel A). (C) Absolute copy numbers of DUSP5 and DUSP5P1 were calculated based on the vector titrations. Presented are the DUSP5/DUSP5P1 copy numbers ratios (means and standard deviations) of in the indicated sample groups. With the exception of the difference between hematopoietic and non-hematopoietic tumor cells, all other differences are statistically significant (non-malignant vs malignant: p<10^−10^; non-malignant vs. HL: p<10^−8^; Student's t-test;).

**Figure 7:**
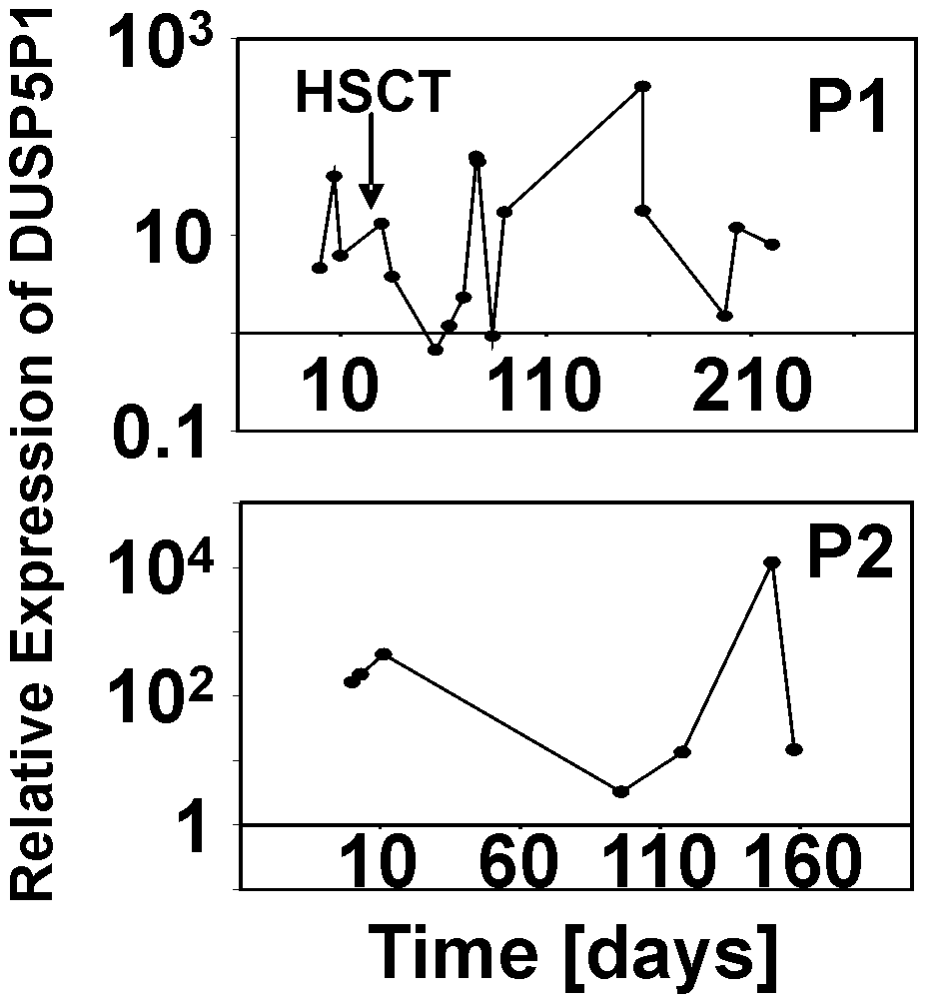
Time course of DUSP5P1 expression in the blood of refractory HL patients. Quantitative RT-PCR was used for determination of expression of DUSP5P1 in PBMCs from two HL patients (P1 and P2). The date of first sampling was set as time point 0. For calculation of relative expression values, GAPDH was used as housekeeping control and the mean Δct value from 5 PBMC samples from healthy donors was set as 1. Patient #1 was treated with hematopoietic stem cell transplantation (HSCT) at the indicated time point.

Quantitative RT-PCR indicated high expression of DUSP5P1 not only in HL cells but also in other tumor cells from hematopoietic and non-hematopoietic malignancies (Figure 6A). In contrast, DUSP5 expression was lower in these cells compared to non-malignant cells (Figure 6A). Again, in most samples the molar concentrations of DUSP5 exceeded the concentrations of DUSP5P1 (Figure 6B and Figure 6C). Only in some of the tumor cell lines this ratio was inverted (samples with values below zero in Figure 6B). We asked whether transcripts corresponding to DUSP5 and DUSP5P1 were detectable in the blood of patients with HL. We analyzed blood samples from two patients with fatal course of HL with quantitative RT-PCR. As shown in Figure 7, both patients showed persistence of a high DUSP5P1 expression over the course of the disease after relapsing.

### The ratio of DUSP5P1 and DUSP5 correlates with expression of B cell leukemia/lymphoma 2-like 11 (BCL2L11)

**Figure 8:**
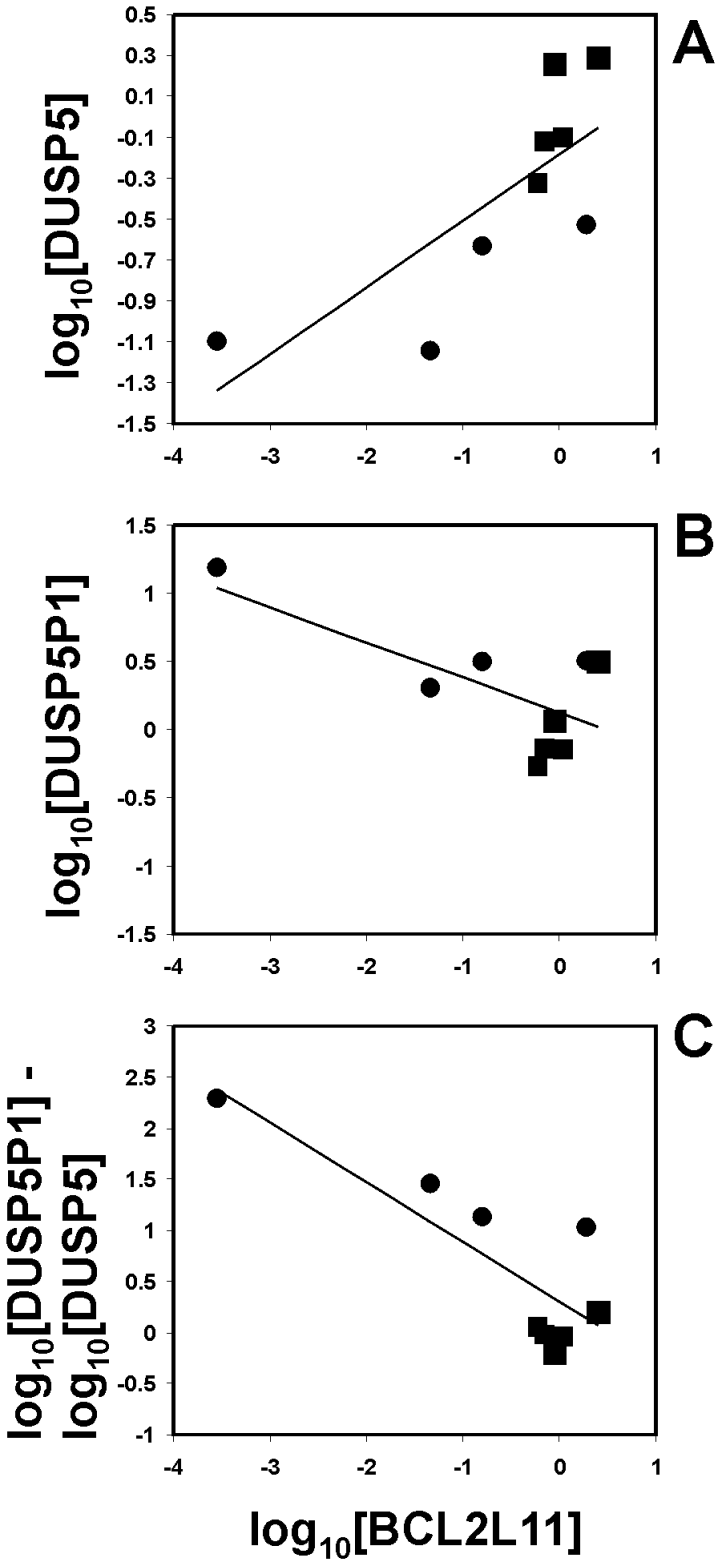
Correlation of DUSP5 and BCL2L11 expression in HL cells and PBMC. Quantitative RT-PCR was used for determination of expression of DUSP5, DUSP5P1, and BCL2L11 in normal PBMCs (squares; N = 5) and HL cell lines (circles; N = 5). For calculation of relative expression values, GAPDH was used as housekeeping control and the mean Δct value from 5 PBMC samples from healthy donors was set as 1. For cell line L-540 no expression of BCL2L11 was detected.

**Figure 9:**
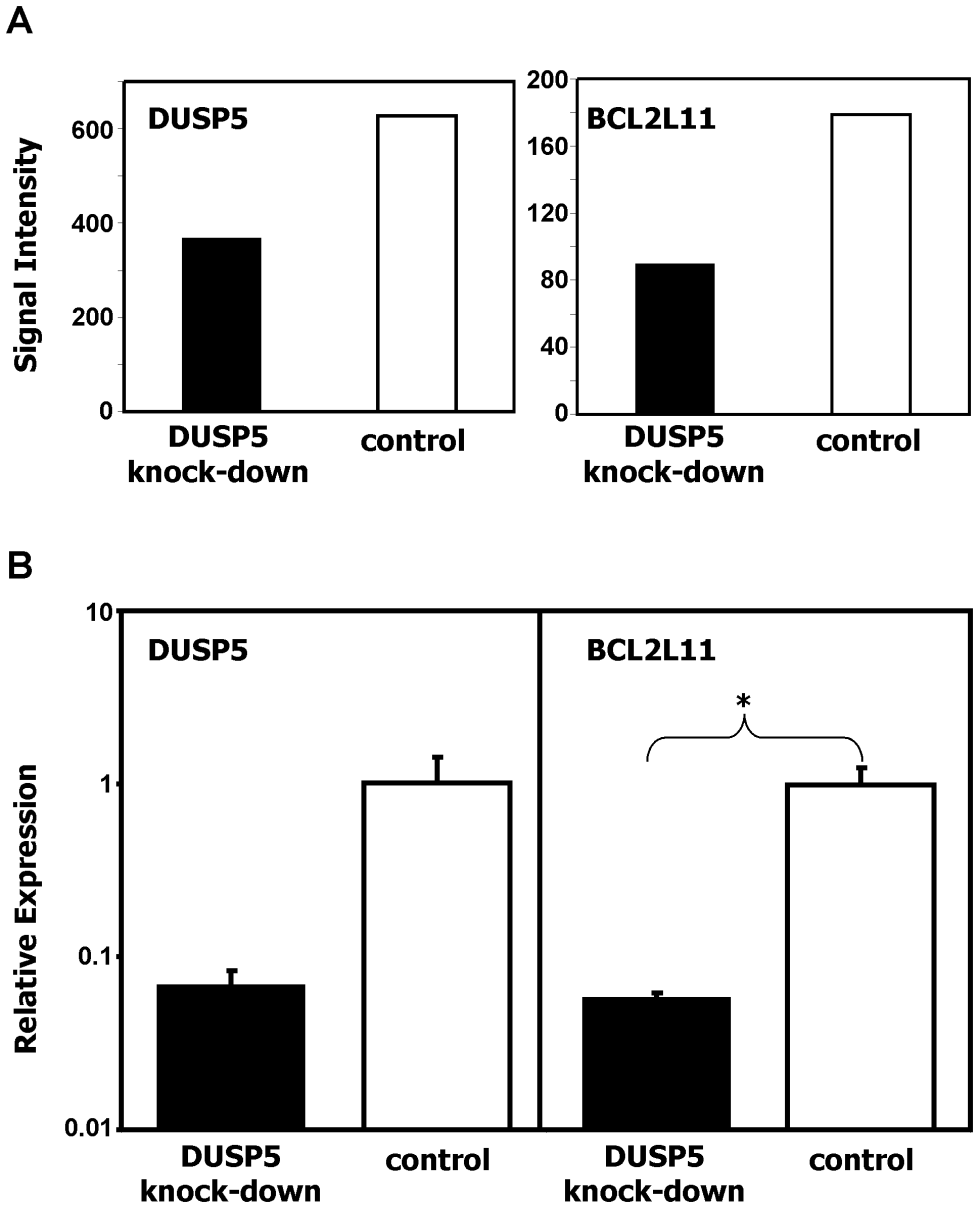
Down-regulation of BCL2L11 after knock-down of DUSP5 in HL cells. Vector based knock-down of DUSP5 in L-428 cells was performed by using the BLOCK-iT POL II miR RNAi expression vector kit as described in Materials and Methods. Gene expression in cells after knock-down of DUSP5 and control cells was analyzed by microarray analysis and quantitative RT-PCR. (A) Results from the microarray analysis. Presented are signal intensities from probe sets corresponding to DUSP5 and BCL2L11 in L-428 cells after knock-down of DUSP5 or after transfection with control vector. (B) Results from RT-PCR analysis. Presented are means and standard deviations from triplicate determinations. For calculation of relative expression values, GAPDH was used as housekeeping control and the median Δct value from control cells was set as 1. Asterisk indicate statistical significance (p<0.05; Student's t-test).

**Figure 10:**
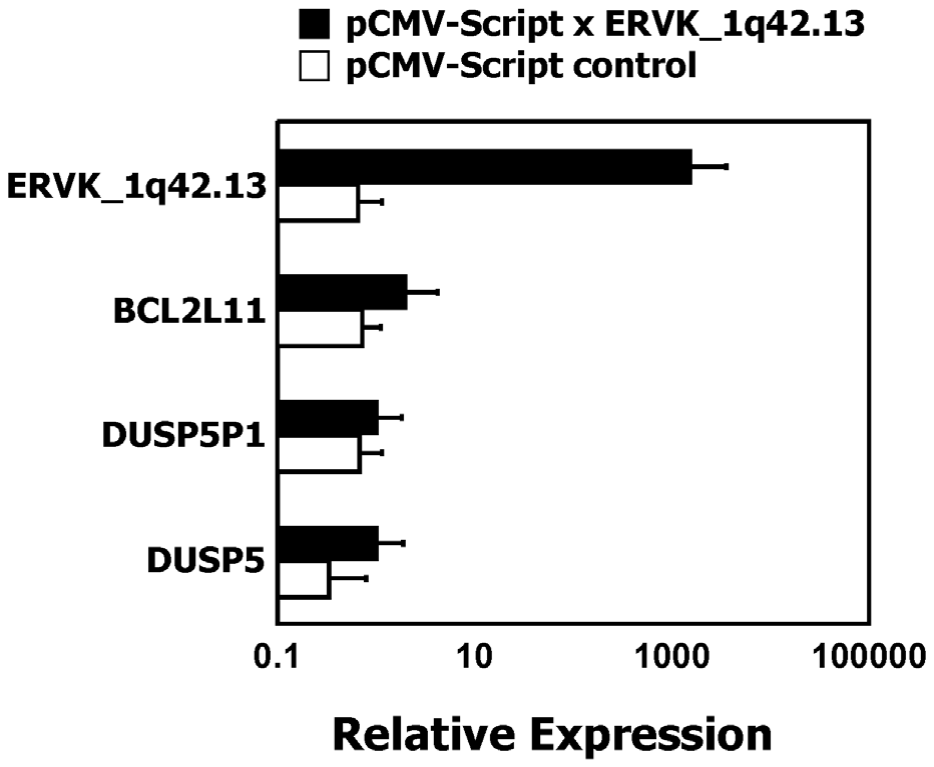
Transgenic over-expression of the short transcript from the cDNA library has no effect on expression of BCL2L11. The vector from the cDNA library with the insert from Figure 1 was used for tranfection of L-428 cells. RNA was isolated 48 h after transfection. Quantitative RT-PCR was used for determination of expression of the transgene (ERVK_1q42.13), BCL2L11, DUSP5P1, and DUSP5, respectively. For calculation of relative expression values, GAPDH was used as housekeeping control and the mean Δct value from control cells was set as 1. Presented are means and standard deviations from 3 independent experiments. With exception of ERVK_1q42.13 (p<10^−6^), all other differences are statistically not significant.

Down-regulation of the pro-apoptotic factor BCL2L11 is a key event after activation of the extracellular signal-regulated kinase (ERK) pathway which is inhibited by members of the dual specificity phosphatase family. We asked whether the expression of DUSP5 and subsequent inhibition of the ERK pathway has an influence on expression of BCL2L11 in HL cells. According to DNA microarray data, HL cells have relatively low signal intensities for BCL2L11 compared to normal cells (Figure S3). Using quantitative RT-PCR, we observed a correlation between high expression of DUSP5 and high expression of BCL2L11 (Figure 8A). Expression of BCL2L11 was high in all tested PBMC samples. HL samples showed lower expression of BCL2L11. In one cell line (L-540) expression of BCL2L11 was completely absent. The expression of DUSP5P1 correlated inversely with the expression of BCL2L11 (Figure 8B). The ratio of DUSP5P1 and DUSP5 was inversely correlated with expression of BCL2L11 (Figure 8C). After knock-down of DUSP5 in HL cell lines, we observed a down-regulation of BCL2L11 in microarray analysis (Figure 9A). Quantitative RT-PCR confirmed down-regulation of BCL2L11 in L-428 cells after knock-down of DUSP5 (Figure 9B). We tested whether over-expression of the short cloned transcript from the cDNA library was able to modulate expression of BCL2L11 in HL cells. As shown in Figure 10, over-expression of this transcript did not suppress expression of DUSP5 or BCL2L11.

## Discussion

HL is a lymphoproliferative disease with unclear pathogenesis. At the molecular level, activation of the nuclear factor of kappa light polypeptide gene enhancer in B-cells (NFKB) signaling pathway has been identified as important mechanism for HL pathogenesis [30]. The human herpesvirus Epstein-Barr virus (EBV) is one factor which can lead to activation of this pathway. EBV is involved in the pathogenesis of several lymphoproliferative diseases [31] and can be found in the tumor cells of a high percentage of HL patients [32]. In addition to EBV, other viruses have been implicated in the pathogenesis of HL. These viruses include measles virus [33], cytomegalovirus [34], torque teno virus (TTV) [35], and human herpesvirus 6 (HHV) [36]. The prevalence of these viruses and EBV in the healthy population is high, indicating that virus infection alone cannot explain lymphoma development. In addition to the mentioned viruses, retroviruses have been implicated in HL pathogenesis [37]. Interestingly, links between putative HL-associated viruses (EBV, HHV6, TTV) and endogenous retroviruses have been established [38]–[42]. EBV and HHV6 can induce expression of endogenous retroviruses [37], [38]. High prevalence of these viruses or immunoreactivity against viral antigens in patients with autoimmune diseases including multiple sclerosis (MS) has been observed [40]–[42]. Epidemiologic similarities between MS and HL have been described and endogenous retroviruses are considered as putative pathogens in MS [13]. Gene expression profiling and immunological analysis of brains from superantigen treated animals further support the concept of the involvement of endogenous retroviruses in the pathogenesis of this disease [43].

Recently, activation of the colony stimulating factor 1 receptor (CSF1R) by activation of an endogenous retrovirus in HL cells has been described [14]. CSF1R is a proto-oncogene and aberrant expression of this receptor might be directly involved in pathogenesis of HL. The observation of expression of ERVK_1q42.13 and DUSP5P1 transcripts in HL cells is another example of linked expression of ERV related sequences and neighboring genes in HL. In contrast to CSF1R, the potential function of DUSP5P1 is unknown. DUSP5P1 is considered to be a pseudogene and no functions of DUSP5P1 transcripts have been described. DUSP5P1 transcripts have been detected in retinoic acid treated NT2 teratocarcinoma cells [44]. Expression of endogenous retroviruses in NT2 cells and other testicular germ cell tumor cell lines is a well-known phenomenon [45]. The presence of ERV sequences in human genes has been shown to confer cell type-specific gene expression [46]. The simultaneous presence of DUSP5P1 transcripts and ERV transcripts in testicular germ cell tumor cells and HL cells suggests that in both cell types transcription is deregulated in a similar way. Together with the epidemiological similarities between HL and testicular cancer [47] this observation might indicate common pathogenetic mechanisms for both diseases.

One possible function of expressed pseudogenes is interference with the activity of the genes from which the pseudogenes are derived. DUSP5P1 is not the only DUSP5 pseudogene in the human genome, suggesting that DUSP5 was frequently involved in genetic rearrangements during human evolution. DUSP5 is involved in T-cell development [48] and alterations in DUSP5 have been observed in lymphoid malignancies [49], [50]. The exact function of DUSP5 in lymphoid cells has to be established. Over-expression of DUSP5 in transgenic mice results in autoimmunity but also in reduced T-cell proliferation [48]. Because DUSP5 is regulated by its substrate mitogen-activated protein kinase 1/extracellular signal-regulated kinase 2 (MAPK1/ERK2) [51], a complex feedback regulation of this pathway might result in signals promoting or inhibiting cell growth and survival. The ERK pathway plays an important role in HL pathogenesis [52]-[54] and inhibition of the negative regulator DUSP5 might be important for proliferation and survival of tumor cells as indicated by the low expression of DUSP5 in the majority of tumor cells. Inhibition of DUSP5 might result in enhanced activation of the ERK pathway and subsequent down-regulation of pro-apoptotic BCL2L11 [55]. DUSP5P1 polypeptides may be able to interfere with the function of DUSP5. Whether DUSP5P1 transcripts were translated into such polypeptides *in vivo* has to be determined. The predicted polypeptides consist only of the regulatory domain of a typical DUSP family member and a catalytic domain will not be present. Only for this domain of DUSP5 the structure has been determined [56]. However, the high sequence homology between DUSP5 and DUSP6 together with the high conservation of amino acids considered to be involved in substrate specificity allows the meaningful modeling of such polypeptide on the basis of DUSP6. It seems unlikely that such DUSP5P1 polypeptides can bind the same substrates as DUSP5, because the amino acids involved in substrate specificity are changed. On the other side, the high homology of the 5′ part of DUSP5P1 and DUSP5 RNAs might allow nonsense-mediated transcriptional gene silencing or activation of other nonsense-mediated decay mechanisms [57]. Other mechanisms include the sequestering of RNA binding molecules which was recently described for non-coding RNA species in patients with amyothrophic lateral sclerosis [58]. In all cases, reduced activity of DUSP5 will result in increased ERK activity and subsequent inhibition of pro-apoptotic BCL2L11. In the future, it might be possible to develop new targeted treatment strategies for HL on the basis of the elucidation of this pathway. The short clone from the cDNA library has no BCL2L11-suppressing activity. This speaks against a simple model in which expression of this sequence inhibit DUSP5 and finally regulate BCL2L11. Relaxed gene regulation in tumor cells can result in generation of transcripts without specific function. On the other hand, we cannot exclude the possibility that other transcripts from the DUSP5P1 locus interfere with expression of function of DUSP5. It seems possible that expression of the ERV sequence is not directly involved in gene regulation but is only a surrogate marker for high transcriptional activity of the complete DUSP5P1 locus in HL cells. The complete spectrum of transcripts form the DUSP5P1/ERVK_1q42.13 locus in HL cells and other tumor cells has not been determined. EBV infection leads to down-regulation of BCL2L11 [59]–[62]. Transgenic over-expression of BCL2L11 isoforms in EBV infected B cells or nasopharyngeal carcinoma cells enhance apoptosis of these cells [59], [60]. BCL2L11 is regulated by the EBV encoded nuclear antigens (EBNA)3A and 3C at multiple levels. EBNA3C can bind directly to the BCL2L11 promoter and epigenetically down-regulates BCL2L11 in conjunction with the polycomb repressive complex 2 [63]. EBNA3C is not expressed in EBV-positive HL and alternative pathways (probably involving DUSP5P1) are required for down-regulation of BCL2L11.

Taken together, we found high expression of DUSP5P1 and low expression of the DUSP5 target BCL2L11 in HL cells. Whether there is a functional connection between these two observations or whether this is only a coincidence requires further investigations. The specificity of DUSP5P1 and ERV transcripts for HL cells might allow the development of methods for detection of minimal residual disease. Circulating HL cells can be present in the peripheral blood of patients with high tumor burden [16]. Our data indicate that DUSP5P1 transcripts are detectable in the peripheral blood of HL patients with active disease. Whether the detection of such HL specific transcripts can be used for the early detection of patients with high risk for relapse should be investigated.

## Supporting Information

Figure S1 **Determination of efficiency of quantitative PCR.** (A) Representative standard curves. Serial 1∶10 dilutions of *Sal*I digested vectors with cloned DUSP5, DUSP5P1, BCL2L11 or GAPDH inserts were used as templates for quantitative PCR with target-specific primers. Linear regression was performed with Microsoft Excel. Dilution 1 corresponds to 1.65 pmol DUSP5P1, 2.32 pmol DUSP5, 0.51 pmol BCL2L11 or 0.50 pmol GAPDH target/tube; dilution 2 corresponds to 165 fmol DUSP5P1, 232 fmol DUSP5, 50.8 fmol BCL2L11 or 50 fmol GAPDH target/tube; dilution 3 corresponds to 16.5 fmol DUSP5P1, 23.2 fmol DUSP5, 5.08 fmol BCL2L11 or 5.04 fmol GAPDH target/tube; dilution 4 corresponds to 1.65 fmol DUSP5P1, 2.32 fmol DUSP5, 508 attomol BCL2L11 or 504 attomol GAPDH target/tube; dilution 5 corresponds to 165 attomol DUSP5P1, 232 attomol DUSP5, 50.8 attomol BCL2L11 or 50.4 attomol GAPDH target/tube. (B) The slopes m of the curves were used for calculation of efficiencies E according to E = 10^−1/m^−1. Presented are means and standard deviations from 2 independent experiments.
(TIF)

Figure S2 **Expression of DUSP5P1 and DUSP5 in HL cell lines.** Presented are results from a quantitative RT-PCR (means and standard deviations from 3 experiments) with primers with specificity for DUSP5 and DUSP5P1. cDNA from HL cell lines and normal PBMC was used as template for PCR. For calculation of expression values, molar amounts of transcripts were calculated on the basis of the standard curves in Figure S1. Data were presented as mol target/mol GAPDH.
(TIF)

Figure S3 **Expression of DUSP5, DUSP5P1 and BCL2L11 in HL cells and normal tissues.** Presented are signal intensities form DNA microarray data form HL samples (red bars) and a panel of normal samples from the Gene Expression Omnibus data base (http://www.ncbi.nlm.nih.gov/gds). The following data sets were used (from left to right): naïve B cells: GSM312870, GSM312872, GSM312874, GSM312875, GSM312876; memory B cells: GSM312877, GSM312879, GSM312882, GSM312883, GSM312886; centrocytes: GSM312887, GSM312890, GSM312893, GSM312894, GSM312895; centroblasts: GSM312937, GSM312938, GSM312939, GSM312940, GSM312941; plasma cells: GSM312942, GSM312943, GSM312944, GSM312945, GSM312946; ovary: GSM175789, GSM176131; breast: GSM175792, GSM175795; synovial membrane: GSM175810, GSM175811; heart atrium: GSM175814, GSM175815; heart ventricle: GSM175817, GSM175819; coronary artery: GSM175820, GSM175821; stomach cardiac: GSM175822, GSM175823; dorsal root ganglia: GSM175825, GSM175827; ventral tegmental area: GSM175829, GSM175831; cervix GSM175833, GSM176130; omental adipose tissue: GSM175834, GSM175836; nipple cross section: GSM175838, GSM175840; amygdala: GSM175842, GSM175844; putamen: GSM175846, accumbens: GSM175849, GSM175851; cerebellum GSM175852, GSM176157; corpus callosum: GSM175855, GSM175857; frontal lobe: GSM175859, GSM175860; hippocampus: GSM175861, GSM175987; parietal lobe: GSM175862, GSM175864; spinal cord: GSM175865, GSM175867; subthalamic nucleus: GSM175869, GSM175870, substantia nigra: GSM175871, GSM175873; temporal lobe: GSM175874; GSM175876; vagina: GSM175878, GSM176129; saphenous vein: GSM175879, GSM175880; skeletal muscle: GSM175882, GSM175883; thalamus: GSM175885, GSM175887; trigeminal ganglia: GSM175889, GSM175891; superior vestibular nuclei: GSM175893, GSM175894; tongue superior with papillae: GSM175896, GSM175898; tongue main corpus: GSM175900, GSM176014; midbrain: GSM175901, GSM175903; prostate GSM175923, GSM175924; thymus gland: GSM175973, GSM176262; bone marrow: GSM175974, GSM176300; trachea: GSM175980, GSM175981; small intestine jejunum: GSM175982; colon cecum: GSM175983; GSM175984; skin: GSM175993; putamen: GSM176020; mammary gland: GSM176231, GSM176232; fallopian tube: GSM176239; aorta: GSM176263, GSM176264; small intestine jejunum: GSM176265; skin: GSM176267, joint tissue synovium: GSM176268, GSM176269; penis: GSM176270, GSM176271; human umbilical vein endothelial cells (HUVEC): GSM176298, GSM176299; deltoid muscle: GSM176301, GSM176312; substantia nigra pars compacta: GSM176393, GSM176401; substantia nigra reticulata: GSM176395, GSM176402; internal gloubus pallidum: GSM176436, GSM176445; external gloubus pallidum: GSM176447, GSM176448; thalamus subthalamic nucleus: GSM176451, GSM176453; thalamus lateral nuclei: GSM176452, GSM176454; bronchus: GSM80578, GSM80579; subcutaneous adipose tissue: GSM80589, GSM80590; adrenal gland cortex: GSM80606, GSM80607; cerebral cortex: GSM80651, GSM80652; endometrium: GSM80672, GSM80673; kidney cortex: GSM80687, GSM80688; hypothalamus: GSM80691, GSM80692; esophagus: GSM80695, GSM80696; lung: GSM80707, GSM80712; medulla: GSM80709, GSM80711; myometrium: GSM80718, GSM80719; liver: GSM80729, GSM80730; kidney medulla: GSM80732, GSM80733; lymph nodes: GSM80736, GSM80737; pharyngeal mucosa: GSM80748, GSM80749; nodose nucleus: GSM80769, GSM80770; occipital lobe: GSM80773, GSM80774; oral mucosa: GSM80777, GSM80778; stomach fundus: GSM80810, GSM80811; stomach pyloric: GSM80814, GSM80815; pituitary gland: GSM80817, pituitary gland: GSM80818; salivary gland: GSM80821, GSM80822; spleen: GSM80825, GSM80826; thyroid gland: GSM80865, GSM80866; tonsil: GSM80886, GSM80889; vulva: GSM80897,GSM80898; urethra: GSM80911, GSM80912; testes: GSM176276, GSM176422, GSM176423, GSM176275, GSM176113; L-428 cells: GSM311200, GSM499721, GSM499729, GSM637960; L-1236 cells: GSM499722, GSM499730, GSM637962; KM-H2 cells: GSM499723, GSM311194, GSM499731, GSM637959; HDLM-2 cells: GSM499724, GSM499732, GSM637963; L-540Cy cells: GSM499726; L-540 cells: GSM637961, GSM499725; L428 cells: GSM1006383, GSM1006384; microdissected HL cells: GSM956644, GSM956645, GSM956646, GSM956647,GSM956648, GSM956649, GSM956650, GSM956651, GSM956652, GSM956653, GSM956654, GSM956655, GSM956656, GSM956657, GSM956658, GSM956659, GSM956660, GSM956661, GSM956662, GSM956663, GSM956664, GSM956665, GSM956666, GSM956667, GSM956668, GSM956669, GSM956670, GSM956671, GSM956672 (Brune V, Tiacci E, Pfeil I, Döring C, Eckerle S et al. (2008) Origin and pathogenesis of nodular lymphocyte-predominant Hodgkin lymphoma as revealed by global gene expression analysis. J Exp Med 205: 2251–2268; Roth RB, Hevezi P, Lee J, Willhite D, Lechner SM, et al. (2006) Gene expression analyses reveal molecular relationships among 20 regions of the human CNS. Neurogenetics 7: 67–80; Liu TY, Wu SJ, Huang MH, Lo FY, Tsai MH, Tsai CH et al. EBV-positive Hodgkin lymphoma is associated with suppression of p21cip1/waf1 and a worse prognosis. Mol Cancer 2010; 9: 32; Köchert K, Ullrich K, Kreher S, Aster JC, Kitagawa M, et al. (2011) High-level expression of Mastermind-like 2 contributes to aberrant activation of the NOTCH signaling pathway in human lymphomas. Oncogene 30: 1831–40; Steidl C, Shah SP, Woolcock BW, Rui L, Kawahara M, et al. (2011) MHC class II transactivator CIITA is a recurrent gene fusion partner in lymphoid cancers. Nature 471: 377–381; Kewitz S, Staege MS (2013) Knock-down of PRAME increases retinoic acid signaling and cytotoxic drug sensitivity of Hodgkin lymphoma cells. PLoS One 8: e55897; Steidl C, Diepstra A, Lee T, Chan FC, Farinha P, et al. (2012) Gene expression profiling of microdissected Hodgkin Reed-Sternberg cells correlates with treatment outcome in classical Hodgkin lymphoma. Blood 120: 3530–3540). All Affymetrix cel files were processed with Affymetrix Expression Console using the MicroarraySuite 5.0 algorithm and scaled to the same target intensity of 500.
(TIF)

Figure S4 **Sequence alignment of DUSP5 and DUSP5 pseudogenes.** DUSP5 pseudogenes were identified by a BLAST search using DUSP5 RNA as query. Presented is a sequence alignment of DUSP5 RNA, the genomic DUSP5 DNA, the genomic DUSP5P1 DNA and 3 newly identified DUSP5 pseudogenes (DUSP5P2, DSUP5Psi3, DSUP5Psi4). The presented sequences correspond to the following database entries: DUSP5-RNA: NM_004419.3, 1–2507; DUSP5: NT_030059.13, 63062089–63075745; DUSP5P1: NT_167186.1, 22303791–22305932; DUSP5P2: NT_025517.18, 9582355–9585843; DUSP5psi3: NT_025028.14, 14042336–14043552; DUSP5psi4: NT_025741.15, 23586534–23586928. Introns of the genomic DUSP5 DNA were indicated by black squares and were not to scale. The lengths of these introns as well as the start position of the 4 exons from DUSP5 are indicated. The pseudogene from chromosome 3 contains additional insertions which are indicated by red triangles. Color code: red: A; green: C; yellow: G; blue: T. Data visualization was performed with GeneDoc (http://www.psc.edu/biomed/genedoc).
(TIF)

Figure S5 **Sequence alignment of the predicted DUSP5P1 peptide and DUSP5 proteins from varying Mammalia.** Presented is an alignment between the putative DUSP5P1 peptide and the homologous region from (predicted) DUSP5 proteins from Oryctolagus anatinus, Monodelphis domesticus, Myotis lucifugus, Bos Taurus, Rattus norvegicus, Canis lupus familiaris, Papio anubis, and Homo sapiens. DUSP5 loci in the genomes of all species were identified essentially as described (Hesse M, Willscher E, Schmiedel BJ, Posch S, Golbik RP, Staege MS (2012) Sequence and expression of the chicken membrane-associated phospholipases A1 alpha (LIPH) and beta (LIPI). Mol Biol Rep 39: 761–769). In all species the DUSP5 gene is located in close proximity to the structural maintenance of chromosomes 3 (SMC3) gene which was used for confirmation that the identified dual specificity phosphatases in all species are DUSP5 and no other members of this gene family (data not shown). Data visualization was performed with GeneDoc (http://www.psc.edu/biomed/genedoc).
(TIF)
