# Supplementary figures and images for: Expression of Dual-Specificity Phosphatase 5 Pseudogene 1 (DUSP5P1) in Tumor Cells
Source: PLoS One. 2014 Feb 24;9(2):e89577. doi: 10.1371/journal.pone.0089577 (PMC3949351; doi:10.1371/journal.pone.0089577)

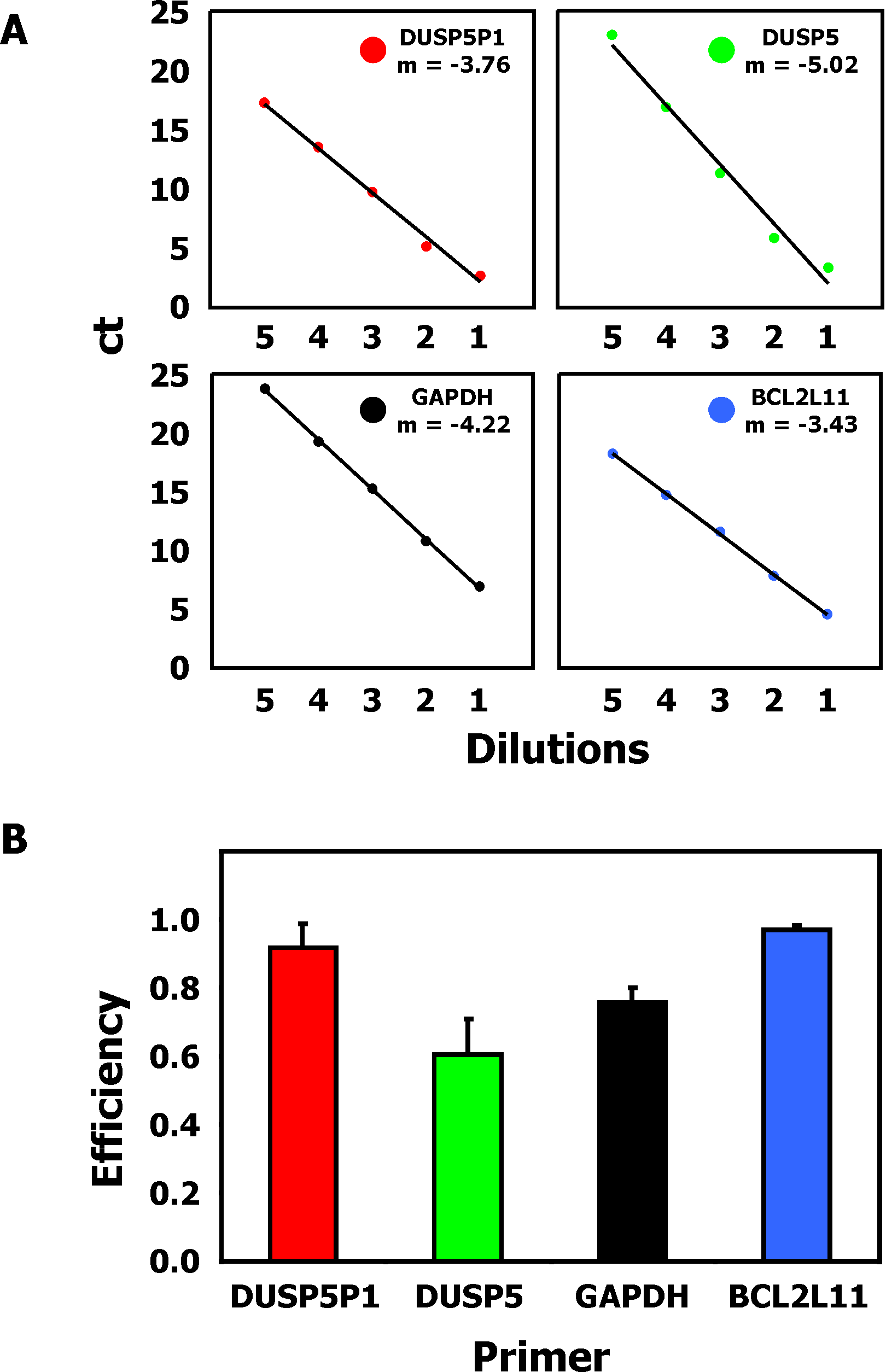

Supplement: Figure S1 — Determination of efficiency of quantitative PCR. (A) Representative standard curves. Serial 1∶10 dilutions of SalI digested vectors with cloned DUSP5, DUSP5P1, BCL2L11 or GAPDH inserts were used as templates for quantitative PCR with target-specific primers. Linear regression was performed with Microsoft Excel. Dilution 1 corresponds to 1.65 pmol DUSP5P1, 2.32 pmol DUSP5, 0.51 pmol BCL2L11 or 0.50 pmol GAPDH target/tube; dilution 2 corresponds to 165 fmol DUSP5P1, 232 fmol DUSP5, 50.8 fmol BCL2L11 or 50 fmol GAPDH target/tube; dilution 3 corresponds to 16.5 fmol DUSP5P1, 23.2 fmol DUSP5, 5.08 fmol BCL2L11 or 5.04 fmol GAPDH target/tube; dilution 4 corresponds to 1.65 fmol DUSP5P1, 2.32 fmol DUSP5, 508 attomol BCL2L11 or 504 attomol GAPDH target/tube; dilution 5 corresponds to 165 attomol DUSP5P1, 232 attomol DUSP5, 50.8 attomol BCL2L11 or 50.4 attomol GAPDH target/tube. (B) The slopes m of the curves were used for calculation of efficiencies E according to E = 10−1/m−1. Presented are means and standard deviations from 2 independent experiments. (TIF) [file pone.0089577.s001.tif]

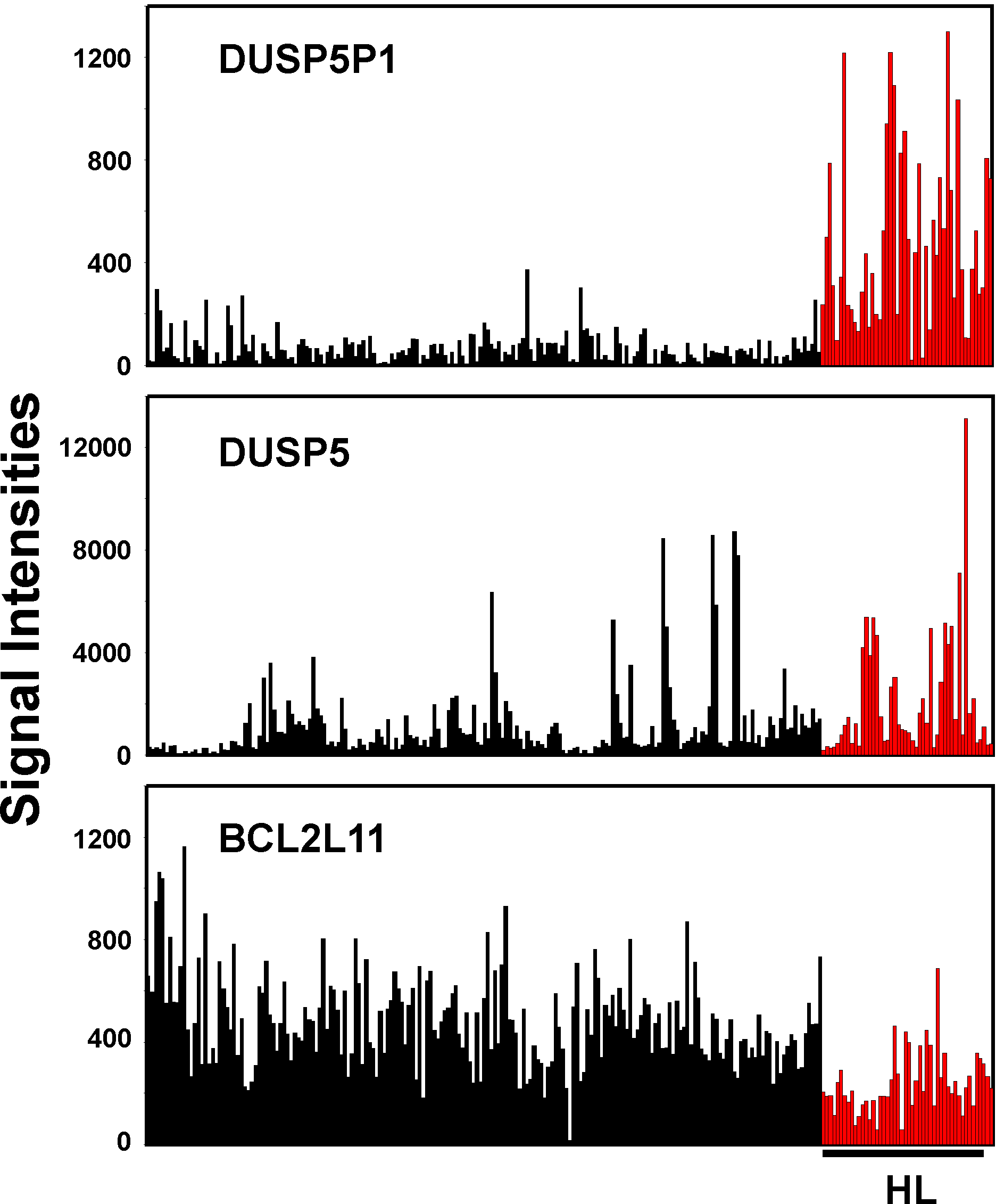

Supplement: Figure S3 — Expression of DUSP5, DUSP5P1 and BCL2L11 in HL cells and normal tissues. Presented are signal intensities form DNA microarray data form HL samples (red bars) and a panel of normal samples from the Gene Expression Omnibus data base (http://www.ncbi.nlm.nih.gov/gds). The following data sets were used (from left to right): naïve B cells: GSM312870, GSM312872, GSM312874, GSM312875, GSM312876; memory B cells: GSM312877, GSM312879, GSM312882, GSM312883, GSM312886; centrocytes: GSM312887, GSM312890, GSM312893, GSM312894, GSM312895; centroblasts: GSM312937, GSM312938, GSM312939, GSM312940, GSM312941; plasma cells: GSM312942, GSM312943, GSM312944, GSM312945, GSM312946; ovary: GSM175789, GSM176131; breast: GSM175792, GSM175795; synovial membrane: GSM175810, GSM175811; heart atrium: GSM175814, GSM175815; heart ventricle: GSM175817, GSM175819; coronary artery: GSM175820, GSM175821; stomach cardiac: GSM175822, GSM175823; dorsal root ganglia: GSM175825, GSM175827; ventral tegmental area: GSM175829, GSM175831; cervix GSM175833, GSM176130; omental adipose tissue: GSM175834, GSM175836; nipple cross section: GSM175838, GSM175840; amygdala: GSM175842, GSM175844; putamen: GSM175846, accumbens: GSM175849, GSM175851; cerebellum GSM175852, GSM176157; corpus callosum: GSM175855, GSM175857; frontal lobe: GSM175859, GSM175860; hippocampus: GSM175861, GSM175987; parietal lobe: GSM175862, GSM175864; spinal cord: GSM175865, GSM175867; subthalamic nucleus: GSM175869, GSM175870, substantia nigra: GSM175871, GSM175873; temporal lobe: GSM175874; GSM175876; vagina: GSM175878, GSM176129; saphenous vein: GSM175879, GSM175880; skeletal muscle: GSM175882, GSM175883; thalamus: GSM175885, GSM175887; trigeminal ganglia: GSM175889, GSM175891; superior vestibular nuclei: GSM175893, GSM175894; tongue superior with papillae: GSM175896, GSM175898; tongue main corpus: GSM175900, GSM176014; midbrain: GSM175901, GSM175903; prostate GSM175923, GSM175924; thymus gland: GSM175973, GSM176262; bone marrow: GSM175974, G [file pone.0089577.s003.tif]

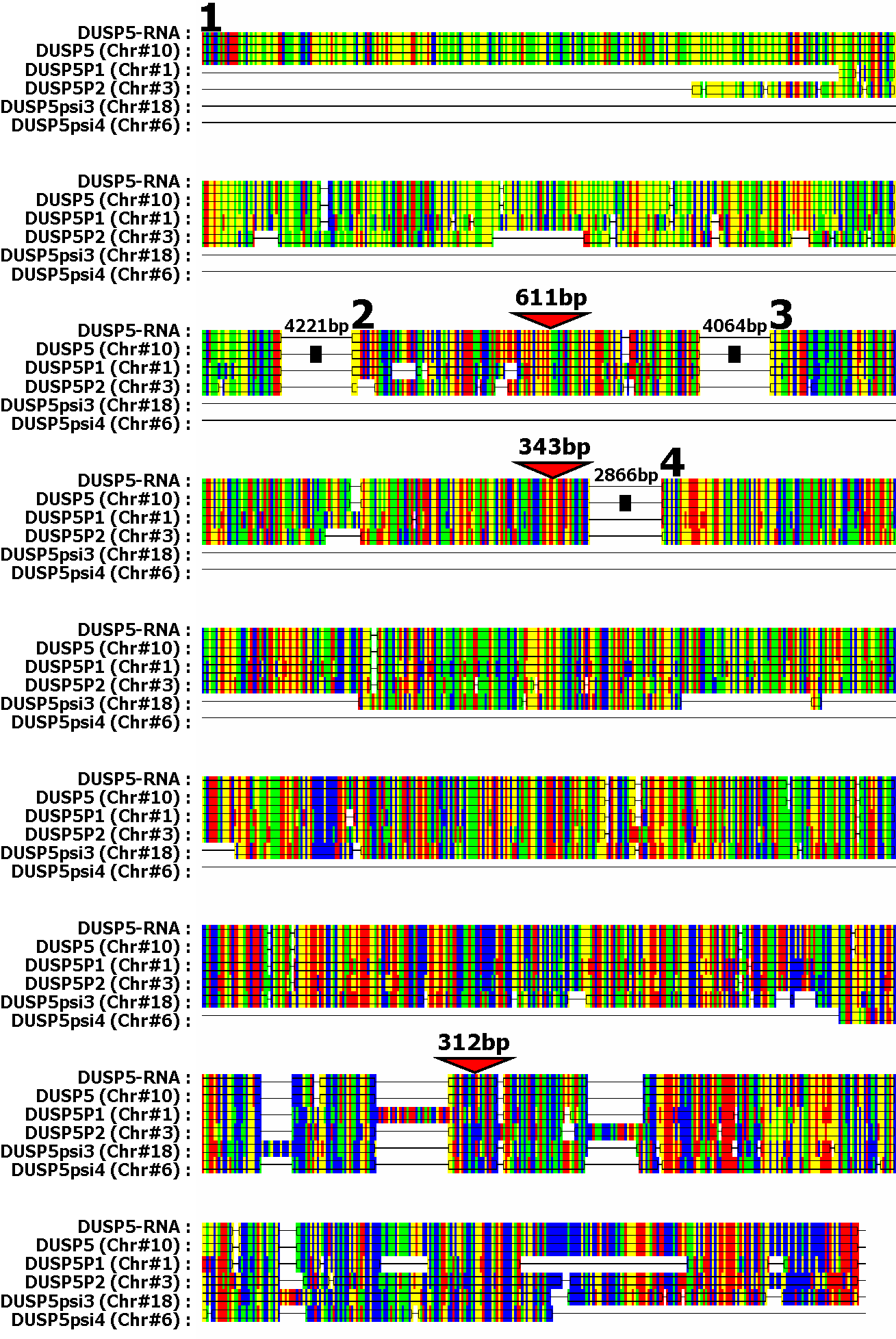

Supplement: Figure S4 — Sequence alignment of DUSP5 and DUSP5 pseudogenes. DUSP5 pseudogenes were identified by a BLAST search using DUSP5 RNA as query. Presented is a sequence alignment of DUSP5 RNA, the genomic DUSP5 DNA, the genomic DUSP5P1 DNA and 3 newly identified DUSP5 pseudogenes (DUSP5P2, DSUP5Psi3, DSUP5Psi4). The presented sequences correspond to the following database entries: DUSP5-RNA: NM_004419.3, 1–2507; DUSP5: NT_030059.13, 63062089–63075745; DUSP5P1: NT_167186.1, 22303791–22305932; DUSP5P2: NT_025517.18, 9582355–9585843; DUSP5psi3: NT_025028.14, 14042336–14043552; DUSP5psi4: NT_025741.15, 23586534–23586928. Introns of the genomic DUSP5 DNA were indicated by black squares and were not to scale. The lengths of these introns as well as the start position of the 4 exons from DUSP5 are indicated. The pseudogene from chromosome 3 contains additional insertions which are indicated by red triangles. Color code: red: A; green: C; yellow: G; blue: T. Data visualization was performed with GeneDoc (http://www.psc.edu/biomed/genedoc). (TIF) [file pone.0089577.s004.tif]

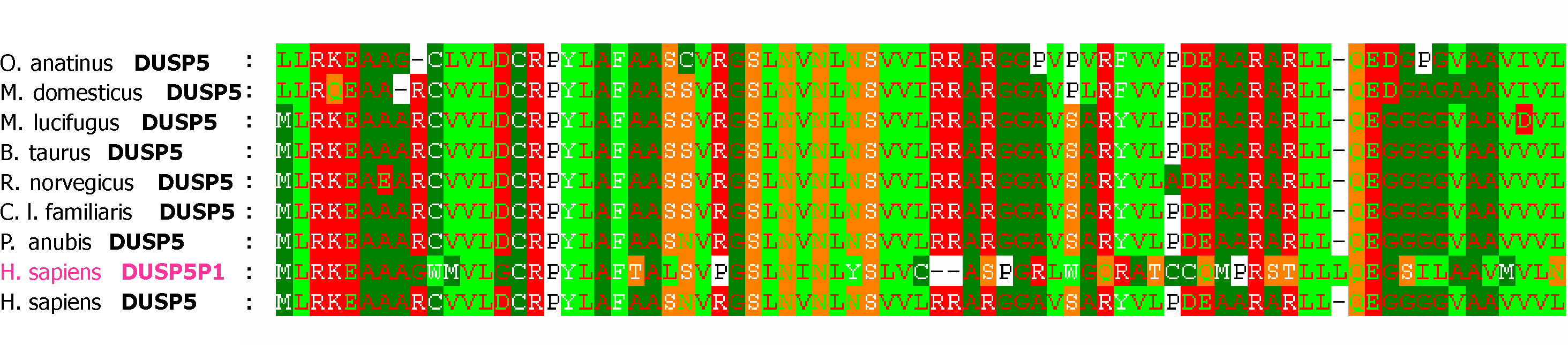

Supplement: Figure S5 — Sequence alignment of the predicted DUSP5P1 peptide and DUSP5 proteins from varying Mammalia. Presented is an alignment between the putative DUSP5P1 peptide and the homologous region from (predicted) DUSP5 proteins from Oryctolagus anatinus, Monodelphis domesticus, Myotis lucifugus, Bos Taurus, Rattus norvegicus, Canis lupus familiaris, Papio anubis, and Homo sapiens. DUSP5 loci in the genomes of all species were identified essentially as described (Hesse M, Willscher E, Schmiedel BJ, Posch S, Golbik RP, Staege MS (2012) Sequence and expression of the chicken membrane-associated phospholipases A1 alpha (LIPH) and beta (LIPI). Mol Biol Rep 39: 761–769). In all species the DUSP5 gene is located in close proximity to the structural maintenance of chromosomes 3 (SMC3) gene which was used for confirmation that the identified dual specificity phosphatases in all species are DUSP5 and no other members of this gene family (data not shown). Data visualization was performed with GeneDoc (http://www.psc.edu/biomed/genedoc). (TIF) [file pone.0089577.s005.tif]
